# Supplementary figures and images for: Two New Sexual Talaromyces Species Discovered in Estuary Soil in China
Source: J Fungi (Basel). 2021 Dec 31;8(1):36. doi: 10.3390/jof8010036 (PMC8778840; doi:10.3390/jof8010036)

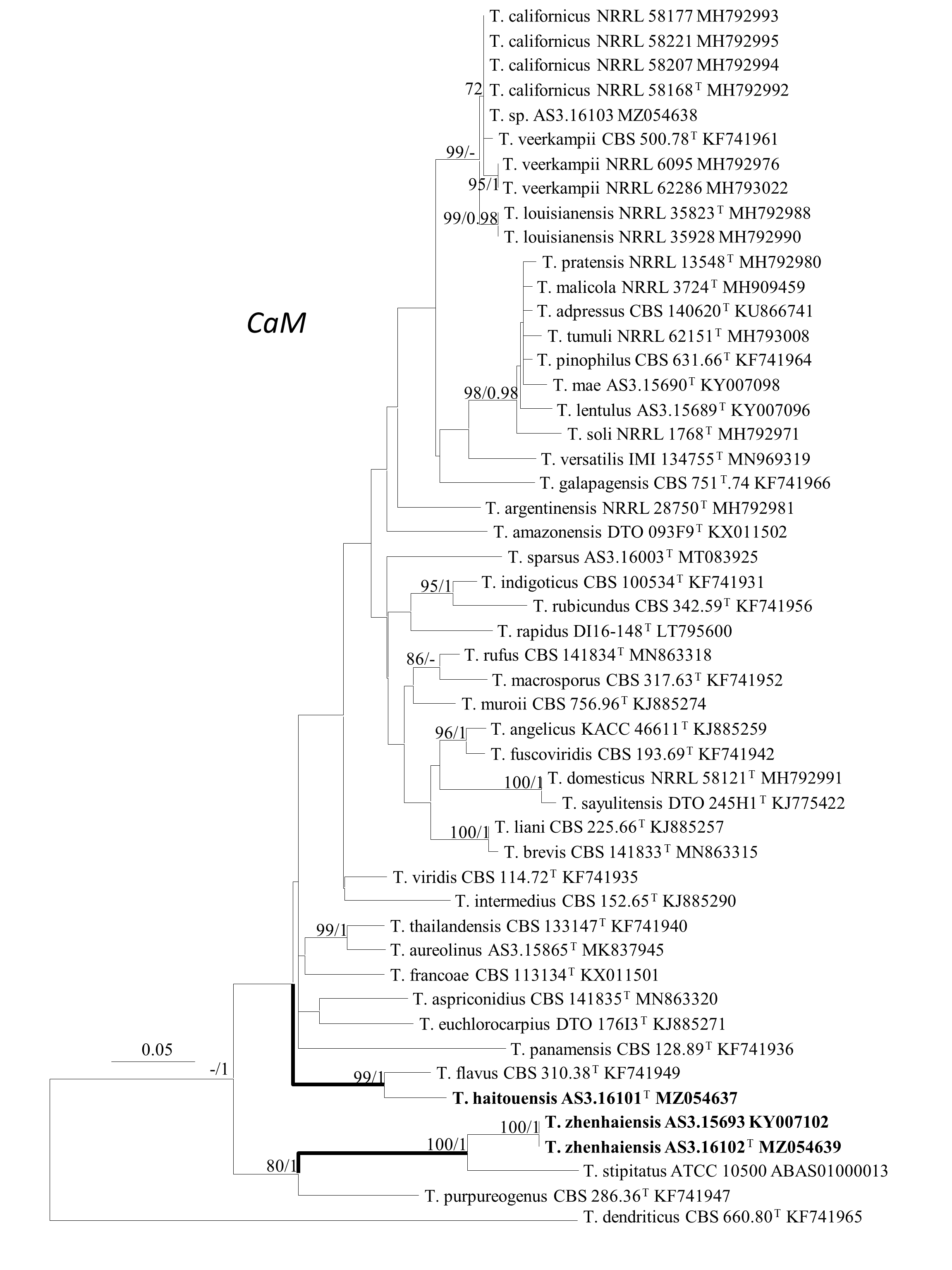

Supplement: Supplementary file 1 [file jof-08-00036-s001.zip › Figure S1.tif]

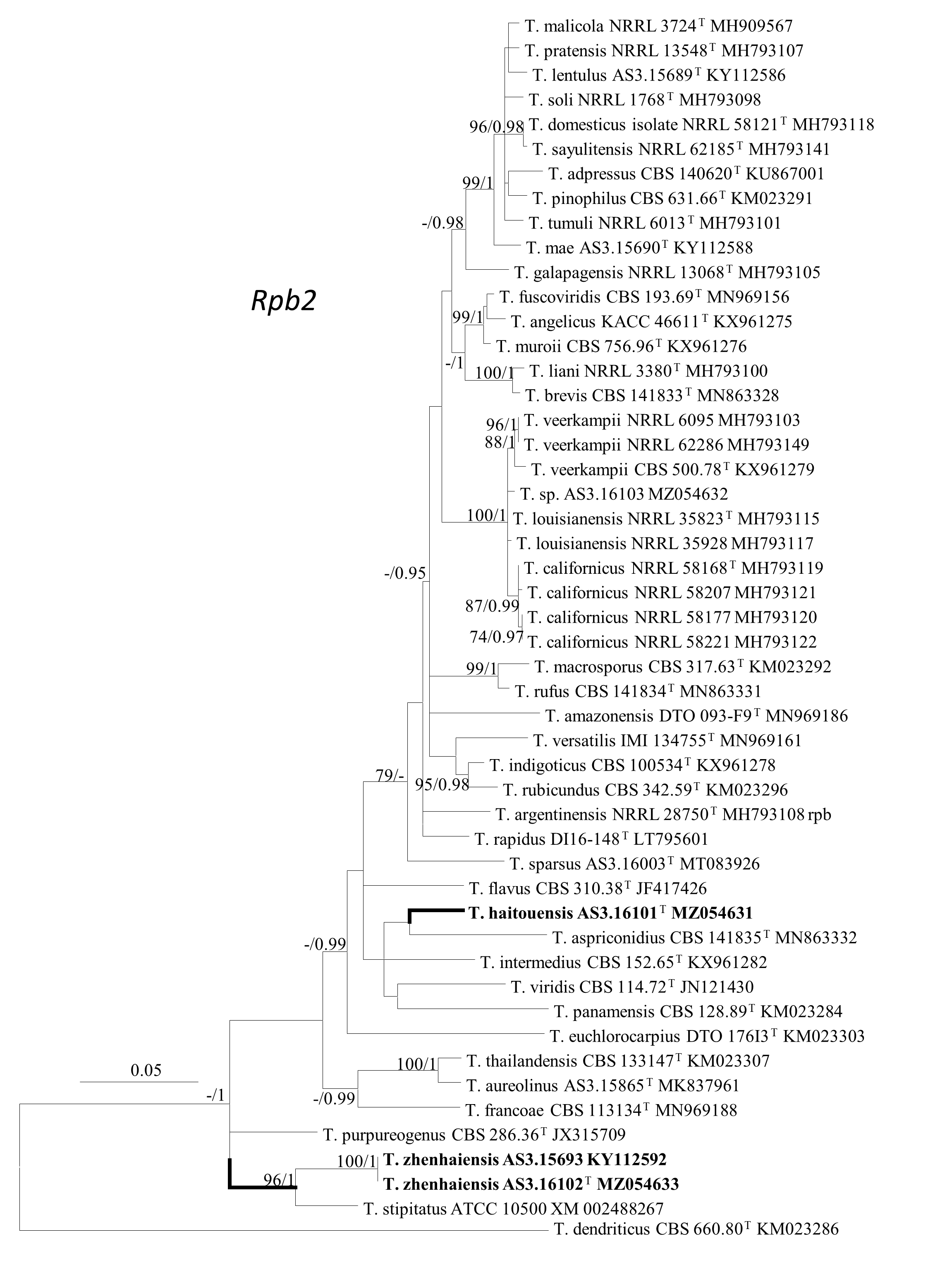

Supplement: Supplementary file 1 [file jof-08-00036-s001.zip › Figure S2.tif]

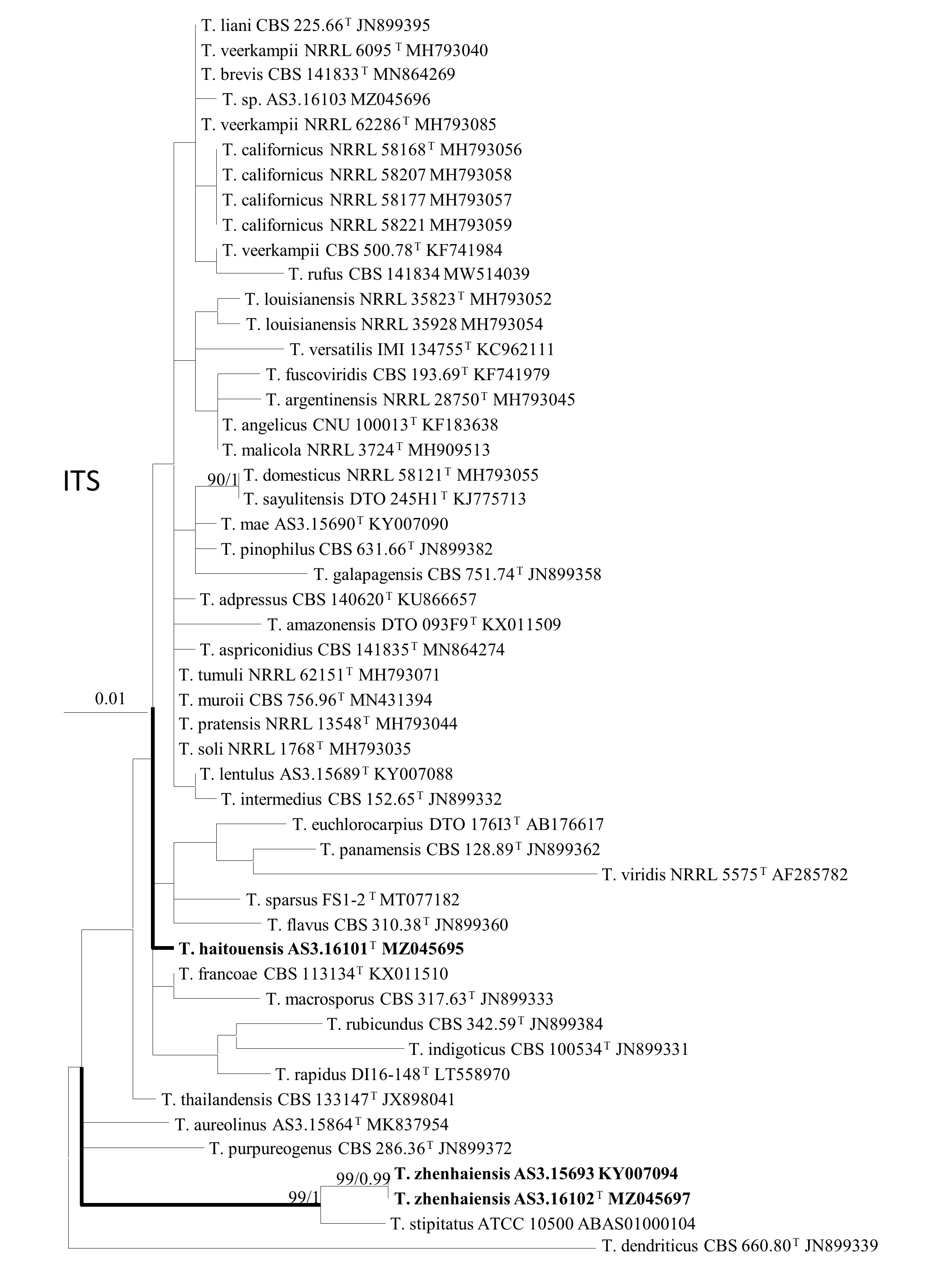

Supplement: Supplementary file 1 [file jof-08-00036-s001.zip › Figure S3.tif]
